# Long-Term Outcomes of Cryoablation for Solitary De Novo T1a Renal Cell Carcinoma: A Tertiary Cancer Center Experience

**DOI:** 10.3390/cancers18203292

**Published:** 2026-10-13

**Authors:** Mohamed E. Abdelsalam, Ahmed Awad, Robert J. Raymond, Zhongya Wang, David Irwin, Peiman Habibollahi, Zeyad A. Metwalli, Stephen R. Lee, Bruno C. Odisio, Thomas Lu, Brittney H. Cotta, Jose A. Karam, Surena F. Matin, Kamran Ahrar

**Affiliations:** 1 Department of Interventional Radiology, The University of Texas MD Anderson Cancer Center, 1515 Holcombe Boulevard, Unit 1471, Houston, TX 77030, USA (A.A.);; 2 Department of Biostatistics, The University of Texas MD Anderson Cancer Center, Houston, TX 77030, USA; 3 Department of Urology, The University of Texas MD Anderson Cancer Center, Houston, TX 77030, USA

**Keywords:** ablation, outcomes, survival rates, renal cell carcinoma

## Abstract

**Objective:** To assess the long-term oncologic and survival outcomes of percutaneous cryoablation (PCA) for patients with solitary de novo T1a renal cell carcinoma (RCC). **Methods:** This retrospective study was conducted following the STROBE guidelines. We included patients with a single, biopsy-confirmed, treatment-naïve T1a RCC (<4 cm) who underwent PCA between January 2008 and December 2021. Patient demographics, tumor characteristics, and procedural details were collected. Survival analyses were performed using the Kaplan–Meier estimator. **Results:** Eighty-six patients (57 men and 29 women) with a median age of 69 years met the inclusion criteria. Fifty patients (58%) had a history of an additional non-renal malignancy. In total, 86 renal tumors were treated using PCA, with a median tumor diameter of 2.7 cm. Clear cell RCC accounted for 67.4% of the tumors. Procedural complications classified as Clavien–Dindo grade III or higher occurred in 4.6% of cases. Local tumor recurrence at the ablation site was observed in five patients (5.8%), and four patients (4.6%) developed new lesions elsewhere within the kidney. The estimated median overall survival was 10.34 years. The 10-year local recurrence-free and disease-free survival rates were 93.5% and 84.0%, respectively. Both metastasis-free survival and cancer-specific survival rates at 10 years were 100%. **Conclusions:** Percutaneous cryoablation provided durable local tumor control with low major morbidity and favorable long-term oncologic outcomes in this selected cohort of patients with solitary cT1a renal tumors.

## Simple Summary

In this study, we evaluated the long-term outcomes of 86 patients with newly diagnosed, biopsy-confirmed kidney cancer smaller than 4 cm who underwent percutaneous cryoablation (extreme cold to destroy the tumor) between 2008 and 2021. We found that serious complications were uncommon, and only a small number of patients developed tumor recurrence. Most patients remained free of cancer over long-term follow-up, with no deaths caused by kidney cancer observed at 10 years. These findings support percutaneous cryoablation as a safe and durable treatment option for selected patients with small kidney cancer.

## 1. Introduction

The need to maintain renal function has driven increased interest in minimally invasive modalities for managing small renal masses (SRMs) [1,2,3,4,5]. Current clinical guidelines from the American Urological Association (AUA) advocate for nephron-sparing approaches, with partial nephrectomy as the preferred intervention, reserving radical nephrectomy for specific clinical indications [2]. Nevertheless, surgical management may not be feasible for all patients, either due to medical comorbidities or personal preference to avoid perioperative risks. In such contexts, image-guided percutaneous thermal ablation (TA) has emerged as a viable, less invasive therapeutic alternative [1,2,5]. Preservation of renal parenchyma is particularly critical in individuals with bilateral tumors or hereditary syndromes—such as Von Hippel–Lindau disease—that predispose to multifocal renal cell carcinoma (RCC). TA facilitates local tumor control while preserving renal function, and it is associated with a lower complication profile, reduced hospitalization times, and suitability for outpatient treatment when compared with traditional surgical methods [4]. Mounting evidence supports the oncologic effectiveness and favorable survival outcomes of TA, leading to endorsement by several professional societies [1,2,3,5]. Since 2017, the AUA has included TA among the recommended treatment options for select patients with renal tumors less than 3 cm in diameter [1].

Percutaneous cryoablation (PCA) was initially adopted for patients with RCC who were not considered surgical candidates. Since then, studies have reinforced its safety and efficacy in the short and intermediate terms [6,7,8,9,10,11,12,13,14,15]. Data on oncologic and survival outcomes extending beyond five years remain limited [16,17,18]. Furthermore, many published studies have included heterogeneous patient populations, incorporating patients with multiple renal tumors, recurrent disease, or lesions without histologic confirmation, potentially limiting accuracy of the results and the interpretation of long-term oncologic outcomes. To address these limitations, the present study evaluates a well-defined cohort of patients with solitary, de novo, biopsy-confirmed T1a RCC treated with PCA. By focusing on this relatively homogeneous population, our study aims to provide a more precise assessment of long-term local tumor control, metastatic progression, and survival outcomes (5 years and 10 years), thereby contributing additional evidence regarding the durability of PCA as a definitive treatment for localized T1a RCC.

## 2. Materials and Methods

### 2.1. Patient Selection

This retrospective observational cohort study was designed and reported in strict adherence to the Strengthening the Reporting of Observational Studies in Epidemiology (STROBE) guidelines. A retrospective review was performed of our institutional renal ablation registry, encompassing patients from January 2008 through to December 2021. The study protocol received approval from the Institutional Review Board, with informed consent requirements waived.

Eligible participants included patients presenting with solitary, de novo, biopsy-proven, treatment-naïve cT1a renal cell carcinoma (RCC) measuring less than 4 cm in maximal diameter who underwent percutaneous image-guided cryoablation. Exclusion criteria included patients with tumors larger than 4 cm, hereditary RCC syndromes (e.g., Von Hippel–Lindau), a prior history of RCC, metastatic disease at the time of presentation, multifocal renal tumors, lesions not biopsy-proven RCC or benign lesions. Patients treated with radiofrequency or microwave ablation were also excluded.

All cases were referred for consideration of ablation following multidisciplinary evaluation initiated by the attending urologist. Eligible patients were subsequently assessed in an outpatient clinic by an interventional radiologist, with review of clinical history, imaging studies, and relevant laboratory investigations.

### 2.2. Ablation Technique

Our approach to ablation has been described in previous publications [19,20,21]. Briefly, at our institution, cryoablation is the preferred method for renal tumors larger than 3.5 cm, conducted under general anesthesia using either computed tomography (CT) or magnetic resonance imaging (MRI) guidance. Tumor size was determined by measuring the maximum tumor diameter on preprocedural most recent cross-sectional imaging (CT or MRI). The number and configuration of cryoprobes were tailored to achieve complete coverage of the tumor with an ice ball margin extending 0.5 to 1.0 cm beyond the tumor margin. The cryoablation protocol consisted of an initial 10-min freeze cycle followed by 8 min of passive thawing and a subsequent 10-min freeze cycle. Active thawing was then performed until the probe temperature reached 25–30 °C. When feasible, tract ablation was performed during probe withdrawal to minimize the risk of bleeding. Histologic confirmation was obtained through percutaneous renal mass biopsy, which was typically performed immediately before ablation during the same procedural session. In a smaller subset of patients with imaging findings that were not sufficiently characteristic of RCC, biopsy was performed in a separate session before the scheduled ablation. Adjunctive maneuvers such as hydrodissection or pyeloperfusion were employed when indicated to protect adjacent structures. For renal lesions not visible on CT, MRI-guided ablation was performed using a 1.5-T MRI scanner (MAGNETOM Espree, Siemens Medical System, Erlangen, Germany) and balanced steady-state free precession techniques for planning and continuous imaging of the kidney [21]. An MRI-compatible cryoablation system was utilized for these procedures, with immediate post-ablation contrast-enhanced cross-sectional imaging performed to evaluate the ablation zone and identify any immediate complications.

Baseline preprocedural renal function was assessed using serum creatinine levels and estimated glomerular filtration rate (eGFR), measured either on the day of ablation or the preceding day.

Patients underwent follow-up clinical evaluations, renal function assessments, and imaging at predefined intervals during the first two years and annually thereafter.

### 2.3. Data Collection

Patient demographics (age and sex), tumor characteristics (size, location, and laterality), histopathology (subtype and Fuhrman grade), history of other malignancies, and procedural details (ablation system used, imaging modality, and use of adjunctive techniques) were retrospectively extracted from the institutional electronic medical record and imaging databases. Data was extracted by independent teams of investigators (led by MEA and AA). In cases of discrepancies between independent reviewers, consensus was reached through discussion with a senior investigator. Cross-sectional imaging studies were reviewed to assess procedural outcomes, including technical success, complications graded according to the Clavien–Dindo classification, residual or recurrent disease, development of new renal tumors outside the treatment zone, and metastatic disease. Survival status, including date and cause of death, was obtained from the electronic medical record and available clinical follow-up data. Missing variables were assessed on a case-by-case basis; patients with missing data for individual variables were retained in the analysis, and the number of available observations was reported for each variable where applicable.

### 2.4. Definitions of Outcomes

Residual disease was defined as persistent contrast enhancement within the ablation zone on the initial follow-up cross-sectional scan. Tumor recurrence referred to newly identified contrast enhancement within or at the margin of the prior ablation zone on subsequent imaging, or histologic evidence of viable malignancy within the treated region. Patients with residual or recurrent disease were considered for additional intervention including repeat ablation, surveillance, or surgical resection.

Oncologic outcome measures were defined according to the American Urological Association (AUA) guidelines for small renal masses [22]: Overall survival (OS) represents the proportion of patients alive at the time of data collection. Local recurrence-free survival (LRFS) refers to the portion of patients without residual or recurrent disease within the ablation site. Metastasis-free survival (MFS) describes patients with no evidence of distant metastatic spread post-ablation. Disease-free survival (DFS) is the proportion of patients with no evidence of RCC disease in either kidney or any distant organ at the last follow-up cross-sectional imaging. Cancer-specific survival (CSS) refers to the proportion of patients who did not die of RCC.

### 2.5. Data Analysis and Statistics

All collected variables were summarized using descriptive statistics. Continuous variables were summarized using means, standard deviations (SDs), medians and ranges for patients with non-missing data and evaluated by Wilcoxon rank-sum test, while categorical variables were summarized using frequencies and percentages and assessed by Fisher’s exact test or its generalization.

Time-to-event outcomes, including OS, LRFS, DFS, MFS, and CSS, were estimated using the Kaplan–Meier survival analysis method. Event timing was calculated from the date of the cryoablation procedure to the date of recurrence (ablation site or kidney), metastasis, or death, depending on the outcome measure.

All the statistical analyses were performed using RStudio version 4.2.2. All statistical tests used a significant level of 5%. No adjustments for multiple testing were performed.

## 3. Results

### 3.1. Patient and Tumor Characteristics

**Figure 1:**
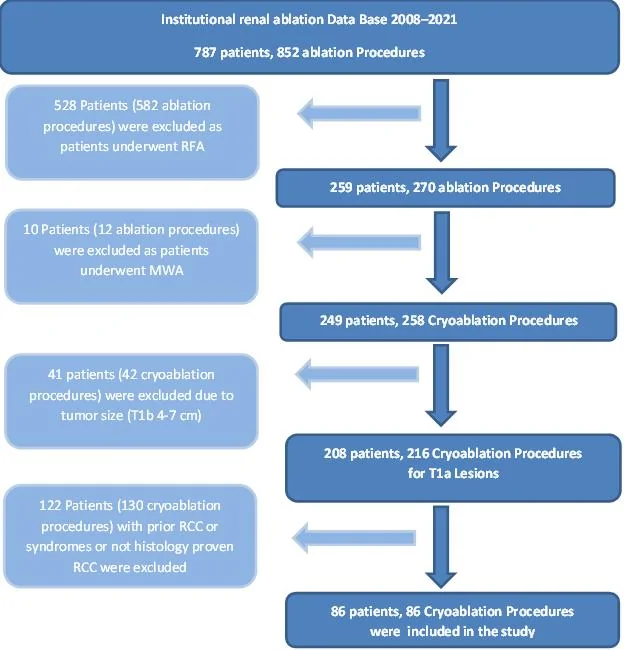
Flowchart showing patients meeting the exclusion criteria for the study.

A total of 86 individuals met the criteria for inclusion. Figure 1 shows a flowchart of patients meeting the inclusion and exclusion criteria for the study. The cohort consisted of 57 males (66.3%) and 29 females (33.7%), with a median age of 69.4 years (range: 28.1–91.3 years). Notably, 58% of patients (n = 50) were concurrently diagnosed with an additional non-renal primary malignancy. Detailed demographic and tumor characteristics are provided in Table 1.

### 3.2. Procedural Outcomes

PCA was performed on 86 renal lesions in 86 patients. The right kidney was involved in 58 cases (67.4%), while the remaining 28 lesions (32.6%) were in the left kidney. The median tumor diameter was 2.7 cm (range: 1.5–4.0 cm). Ablation was performed under CT imaging guidance in 54 procedures (62.8%), and the remaining 32 (37.2%) were performed under MRI guidance. Technical success, defined by adequate probe positioning and completion of the planned ablation, was achieved in all cases. No technical failures were observed in the study cohort. All patients underwent an initial 10-min freeze cycle, followed by 8 min of passive thawing and a second 10-min freeze cycle. Active thawing was subsequently performed until the probe temperature reached 25–30 °C and the probes were removed. Complications classified as Clavien–Dindo grade III or higher were observed in four patients (4.6%). Hemorrhagic events occurred in four cases; three required transarterial embolization, while one developed a blood clot-induced ureteral obstruction necessitating the placement of a ureteral stent. Table 2 outlines all complications, including their classification according to Clavien–Dindo classification and subsequent management.

### 3.3. Histopathological Outcomes

Histological examination of all 86 biopsy-confirmed tumors revealed that clear cell renal cell carcinoma (ccRCC) was the most common subtype, accounting for 67.4% of cases. Papillary RCC comprised 21% of the lesions. Fuhrman grading was available for 72 tumors, with 80% categorized as grade II. A complete breakdown of histologic subtypes and grading is shown in Table 1.

### 3.4. Oncologic Outcomes

Clinical and radiographic surveillance was conducted for all patients, with a mean follow-up duration of 3.71 years (median 2.7 (IQR, 1.5–5) years, range: 0.2–13.5 years).

#### 3.4.1. Residual Disease or Local Recurrence

None of the patients had evidence of residual disease on initial post-ablation imaging. Local recurrence within the treated ablation zone was identified in 5 patients (5.8%), with all events occurring beyond 6 months post-procedure. The median time to recurrence was 15.2 months (range: 9.9–25.6 months). All tumors associated with recurrence were histologically classified as clear cell RCC. Of these five patients, two underwent surveillance and three were managed with repeat ablation. Characteristics of tumor recurrence and management are summarized in Table 3.

#### 3.4.2. Renal Recurrence and Distant Metastases

Recurrence in the kidney (other than the ablation zone) occurred in four patients (4.6%), with a median detection interval of 34.25 months (range: 21–85.4 months). Two patients were managed with additional ablation, while the other two underwent active monitoring: one of these eventually received partial nephrectomies. At the time of data collection, no patient had developed distant metastases from RCC, corresponding to an MFS rate of 100%.

### 3.5. Survival Outcomes

**Figure 2:**
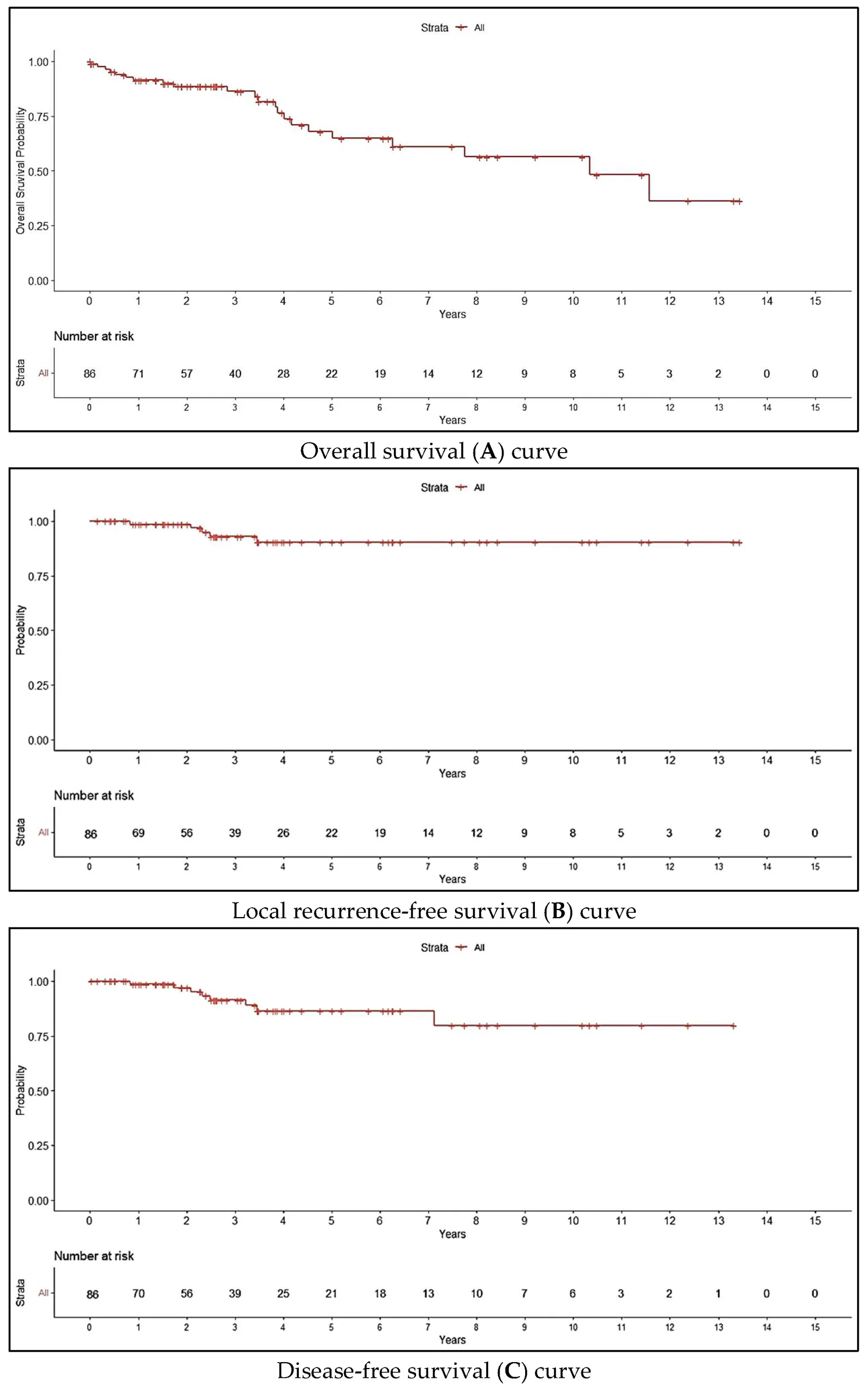
Kaplan–Meier survival curves; overall survival (**A**), local-recurrence-free survival (**B**), and disease-free survival (**C**) curves for the study patients.

The median OS for the study population was 10.34 years. Estimated OS rates at 5 and 10 years were 68.9% and 57.1%, respectively (Table 4). The LRFS rate was 93.5% at both 5 and 10 years, while DFS rates were 92% and 84% at 5 and 10 years, respectively. Both MFS and CSS rates remained at 100%, as no patients experienced systemic disease progression or RCC-related mortality. Kaplan–Meier survival estimates for OS, LRFS, and DFS are illustrated in Figure 2.

## 4. Discussion

This study reports 5-year and 10-year OS rates of 68.9% and 57.1%, respectively, for all patients. The 5-year LRFS, DFS, MFS, and CSS rates were 93.5%, 92%, 100%, and 100%, respectively; the corresponding 10-year rates were 93.5%, 84%, 100%, and 100%. Although these long-term outcomes were estimated using Kaplan–Meier analysis, the 10-year estimates should be interpreted with caution given the progressively decreasing number of patients at risk over time.

In a previously published analysis [20], a statistically significant disparity in OS was observed between patients diagnosed solely with renal cell carcinoma (RCC) and those with concurrent non-renal malignancy. Given that 58% of the present study population had an additional primary cancer, the observed OS outcomes are consistent with these earlier findings. Nonetheless, the median OS for the entire cohort was 10.34 years.

Comparable outcomes have been reported in the literature. Georgiades et al. [11] conducted a prospective single-center study involving 134 patients with biopsy-proven RCC, where the mean tumor size was 2.8 ± 1.4 cm. They observed a 5-year efficacy rate of 97%, defined by stable or non-enhancing lesions on follow-up imaging, and reported both MFS and CSS rates of 100%. These findings align with our study’s 5-year LRFS, MFS, and CSS rates of 93.5%, 100%, and 100%, respectively [11].

In a large retrospective cohort, Breen et al. [6] analyzed 433 patients with 484 renal tumors, including 268 cases of biopsy-confirmed RCC. The median tumor size was 3.4 cm, and the median follow-up period was 31 months (range: 3–99 months). The investigators reported primary and overall ablation efficacy rates of 95.6% and 98.1%, respectively. Among 221 patients with sporadic, biopsy-confirmed RCC, 220 had follow-up imaging extending beyond 3 months. Reported recurrence-free and metastasis-free survival rates were 93.9% and 94.4%, respectively—closely mirroring the 5-year outcomes observed in our study [6].

Gobara et al. [12] evaluated 33 patients with tumors measuring an average of 2.1 ± 0.6 cm (range: 1.0–3.4 cm) and a median follow-up of 60.1 months (range: 18.4–76.6 months). There were no cases of local progression or distant metastasis, and only one death occurred from an unrelated cause 46 months after cryoablation. The CSS remained at 100% at 5 years, which is consistent with our findings [12].

The long-term survival outcomes observed in our cohort are consistent with previously published 5- and 10-year survival data for patients undergoing partial nephrectomy (PN), the standard nephron-sparing surgical approach for patients with localized T1a RCC [22,23]. In a large cohort of 1055 individuals with T1a RCC, Andrews et al. reported 5-year LRFS, MFS, and CSS rates of 97.4%, 98%, and 99.3%, respectively [22]. Similarly, Antonelli et al. evaluated 992 patients treated with PN. For the cT1a subgroup, the authors documented 5-year and 10-year CSS rates of 94.7% and 90.4%, respectively [23]. These findings are comparable to the favorable oncologic outcomes observed in our cohort and further demonstrate the potential for durable local tumor control and excellent CSS following percutaneous cryoablation in appropriately selected patients with T1a RCC. Although direct comparisons between treatment modalities should be interpreted cautiously given differences in patient selection, comorbidity profiles, and tumor characteristics, the sustained long-term outcomes observed in our cohort further support percutaneous cryoablation as an effective nephron-sparing treatment option for selected patients with localized T1a RCC.

Our study has several strengths and limitations. A key strength is the exclusive inclusion of histologically confirmed RCC cases. Furthermore, the extended duration of follow-up enhances the reliability of long-term outcome assessment, with over 25% of patients followed for more than five years. However, the study’s single-center nature may limit its external validity, and the relatively modest sample size restricts the generalizability of findings. Additionally, the retrospective design inherently carries the potential for selection bias and unmeasured confounding. The absence of a control arm further limits the ability to draw direct comparisons between cryoablation and alternative management strategies, such as surgery or surveillance.

## 5. Conclusions

Percutaneous cryoablation provided durable local tumor control with low major morbidity and favorable long-term oncologic outcomes in this selected cohort of patients with solitary cT1a renal tumors.

## Figures and Tables

**Table 1:** Summary of Demographic and Clinical Characteristics—T1a.

	(n = 86)	
Characteristic	N	%
Age		
Mean (SD)	69.2 (10.1)	
Min (Med) Max	28.1 (69.4) 91.3	
Size of lesion		
Mean (SD)	2.7 (0.7)	
Min (Med) Max	1.5 (2.7) 4.0	
Gender		
Female	29	33.7
Male	57	66.3
Left/Right		
Left	28	32.6
Right	58	67.4
Guidance Modality		
CT	53	61.6
CT, US	1	1.2
MRI	32	37.2
Ablation Modality		
Cryoablation	86	100.0
Pathology		
Clear cell	58	67.4
Papillary	18	21.0
Oncocytic neoplasm	3	3.5
Chromophobe	2	2.3
Other subtypes	5	5.8
Fuhrman nuclear grade (n = 72) *		
Grade 1	10	13.8
Grade 2	58	80.6
Grade 3	4	5.6
Indications for Ablation (individual patients may have had multiple indications)		
Medical Comorbidities	37	43
Another non renal malignancy	50	58
Impaired renal functions (GFR < 60 mL/min)	25	29
Patient preference for ablation	6	7

* Fuhrman nuclear grade was not reported in the pathology reports of 14 patients.

**Table 2:** Clavien–Dindo grade III complications and their management.

Complication	Grade	Management
Perinephric hematoma	3b	Angiography and embolization
Hematuria, obstructing clot	4	Double J stent placement and ICU admission
Hematoma/pseudoaneurysm	3b	Angiography and embolization
Perinephric hematoma	4	Angiography and embolization, ICU admission

**Table 3:** Characteristics of ablation zone local recurrences and their management.

Pt	Tumor Size (in cm)	Histology Subtype	Fuhrman Grade	Time to Recurrence (in Months)	Size of Recurrence (in cm)	Ablation Zone Site of Recurrence	Management of Local Recurrence
1	3.6	Clear cell	NR	25.6	1.4	Lateral Margin	surveillance
2	2.6	Clear cell	2	15.2	1.3	Medial margin	RFA
3	3.2	Clear cell	2	17.4	1.1	Medial margin	RFA
4	2.9	Clear cell	2	12.2	1.1	Inferior margin	RFA
5	2.6	Clear cell	1	9.9	0.5	Superior margin	surveillance

**Table 4:** Survival rates of the study patients.

	5-Year Survival, % (95% CI)	10-Year Survival, % (95% CI)
OS	68.9 (56.3–84.2)	57.1 (42.5–76.8)
LRFS	93.5 (82.6–99.0)	93.5 (82.6–99.0)
DFS	92 (77.4–96.6)	84 (65.8–96.7)

OS, overall survival; LRFS, local recurrence-free survival; DFS, disease-free survival.

## Data Availability

The data presented in this study are available on request from the corresponding author due to patient privacy, confidentiality considerations, and institutional restrictions governing the sharing of protected health information.
